# GLADIATOR: a global approach for elucidating disease modules

**DOI:** 10.1186/s13073-017-0435-z

**Published:** 2017-05-26

**Authors:** Yael Silberberg, Martin Kupiec, Roded Sharan

**Affiliations:** 1 Department of Molecular Microbiology and Biotechnology, Tel Aviv University, Tel Aviv, Israel; 2 The Blavatnik School of Computer Science, Tel Aviv University, Tel Aviv, Israel

**Keywords:** Disease gene prediction, Disease modules, Disease pathways, Graphs and networks, Protein-protein interaction network, Hyperinsulinism

## Abstract

**Background:**

Understanding the genetic basis of disease is an important challenge in biology and medicine. The observation that disease-related proteins often interact with one another has motivated numerous network-based approaches for deciphering disease mechanisms. In particular, protein-protein interaction networks were successfully used to illuminate disease modules, i.e., interacting proteins working in concert to drive a disease. The identification of these modules can further our understanding of disease mechanisms.

**Methods:**

We devised a global method for the prediction of multiple disease modules simultaneously named GLADIATOR (GLobal Approach for DIsease AssociaTed mOdule Reconstruction). GLADIATOR relies on a gold-standard disease phenotypic similarity to obtain a pan-disease view of the underlying modules. To traverse the search space of potential disease modules, we applied a simulated annealing algorithm aimed at maximizing the correlation between module similarity and the gold-standard phenotypic similarity. Importantly, this optimization is employed over hundreds of diseases simultaneously.

**Results:**

GLADIATOR’s predicted modules highly agree with current knowledge about disease-related proteins. Furthermore, the modules exhibit high coherence with respect to functional annotations and are highly enriched with known curated pathways, outperforming previous methods. Examination of the predicted proteins shared by similar diseases demonstrates the diverse role of these proteins in mediating related processes across similar diseases. Last, we provide a detailed analysis of the suggested molecular mechanism predicted by GLADIATOR for hyperinsulinism, suggesting novel proteins involved in its pathology.

**Conclusions:**

GLADIATOR predicts disease modules by integrating knowledge of disease-related proteins and phenotypes across multiple diseases. The predicted modules are functionally coherent and are more in line with current biological knowledge compared to modules obtained using previous disease-centric methods.

The source code for GLADIATOR can be downloaded from http://www.cs.tau.ac.il/~roded/GLADIATOR.zip.

**Electronic supplementary material:**

The online version of this article (doi:10.1186/s13073-017-0435-z) contains supplementary material, which is available to authorized users.

## Background

A grand challenge of genetics and medicine is to further our understanding of the molecular basis of disease. Partial collections of disease-related proteins, obtained using traditional and emerging high-throughput technologies, are available in public databases such as the Online Mendelian Inheritance in Man (OMIM) [1] and Genome-Wide Association Studies (GWAS) catalogs [2]. The increasing knowledge regarding these disease-causing genes facilitates the development of new inference methods, harnessing the available information to suggest new candidate causal genes.

Computational methods for associating genes with diseases often employ integrative approaches, exploiting different data sources such as Gene Ontology (GO) annotations [3], protein sequence [4], phenotypic data [5], gene expression data [6, 7], or protein-protein interaction (PPI) information [8, 9]. The latter is increasingly used to reveal novel disease-related proteins based on the observation that genes related to the same disease tend to physically interact in the protein network [9, 10]. For example, Köhler et al. [11] performed a random walk on the PPI network, starting at the known disease genes, and ranking candidate genes by the steady-state probabilities induced by the walk. Xu and Li [12] trained a classifier based on topological network properties to identify genes that are likely to be involved in hereditary diseases. Liu et al. [7] created a statistical framework for constructing a sample-specific network describing an individual’s disease state. The method compared gene expression correlations of a specific cell line to a background set and highlighted all edges in a PPI network in which gene expression correlation was dramatically altered. The resulting subnetwork captured a set of interacting genes which are dysregulated in the disease state. Mazza et al. [13] developed an integer linear programming framework for the prediction of disease-related complexes, based on a seed set of known causal proteins. Their method ranked proteins according to their proximity in a PPI network to the seed proteins. It then identified dense network regions of highly ranked proteins.

A recent study by Menche et al. [14] suggested that disease-causing genes create a connected module in the PPI network and, furthermore, that the distance in the PPI network between disease modules is correlated with their phenotypic similarity. In particular, the study illustrated that disease pairs with overlapping modules exhibit higher phenotypic similarity and co-morbidity values [14]. A follow-up work [15] proposed the DIseAse MOdule Detection (DIAMOnD) algorithm, where the topological structure of the PPI network is used to expand a seed of disease-related proteins into a wider disease module. The method greedily added proteins according to the significance of their connections to the proteins of the growing module, starting from a seed of known proteins. Another approach, suggested by Leiserson et al. [16], attempted to reveal significant cancer modules. Their method, called HotNet2, searched for connected subnetworks by diffusing heat from a seed set of mutated genes; the heat was diffused across the edges of a PPI network until equilibrium was reached. Subnetworks containing nodes that both send and receive a significant amount of heat (strongly connected components) were identified as disease modules.

The use of phenotypic properties to derive disease-associated genes has been facilitated in recent years by the emergence of publicly available disease-phenotype databases. The new wealth of data promotes the examination of molecular mechanisms underlying disease phenotypes. Xu et al. [17] presented an automatic approach to extract disease-phenotype pairs from biomedical literature, obtaining more than 100,000 such pairs. Their study revealed that the number of shared genes between diseases increases directly with the number of shared phenotypes. The human symptoms-disease network [18] utilized biomedical literature to construct a symptom-based human disease network and to study the relation between the clinical phenotypes of a disease and its underlying molecular mechanism. Using this network, the authors demonstrated that disease phenotypic similarity strongly correlates with both shared genetic associations and the extent to which their associated genes interact. The Human Phenotype Ontology (HPO) project [19] annotated thousands of diseases obtained from OMIM [1], Orphanet [20], and the Database of Chromosomal Imbalance and Phenotype in Humans Using Ensembl Resources (DECIPHER) [21] to a structured phenotype ontology. The Phenopolis open data source [22] utilized the HPO database, together with genetic data sources, for both prioritizing disease-causal genes and uncovering gene-phenotype relationships. Freudenberg and Propping [5] devised a ranking algorithm for disease-related genes by combining disease phenotypic similarity, obtained from OMIM, and protein functional similarity, obtained from the GO [23]. Their algorithm clustered diseases according to their phenotypic similarity and then ranked candidate proteins for a disease according to their GO similarity to proteins known to associate with diseases in the corresponding cluster. Wu et al. [24] devised a method that integrates PPI with disease phenotypic similarity to predict disease genes. Their results further demonstrated the global concordance between the PPI network and the phenotypic network. Similarly, Li and Patra [25] constructed a hybrid gene and phenotype network by integrating a gene network and a phenotype network using gene-phenotype relationships. The resulting network was used to prioritize gene-phenotype relations and further to predict disease-disease associations. However, all of the above methods, though they were able to prioritize disease- and phenotypic-related genes, did not provide a coherent connected module in the PPI; thus, their ability to describe disease underlying mechanisms is limited.

**Fig. 1:**
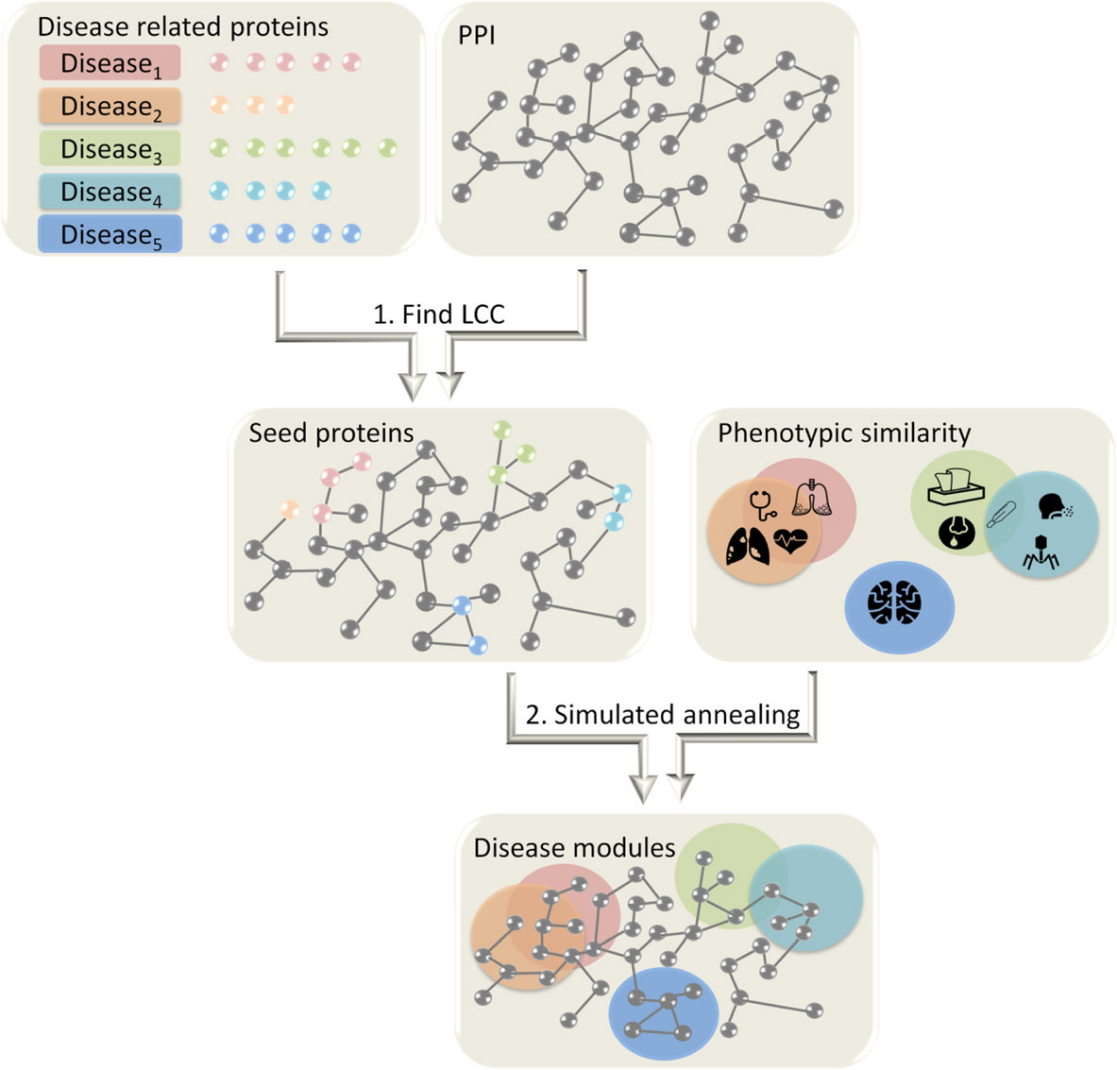
The algorithmic pipeline. Known disease-related proteins (*KnownDisPS*) are projected into the PPI network. The largest connected component (*LCC*) is obtained for each disease. A simulated annealing procedure simultaneously expands all LCCs to disease modules while minimizing the squared Euclidean distance between the module-based similarities of the diseases to a given phenotypic-based similarity

Here, we present GLobal Approach for DIsease AssociaTed mOdule Reconstruction (GLADIATOR), a global approach for module detection. GLADIATOR leverages on phenotypic similarity information and a protein-protein interaction network to predict disease modules for hundreds of diseases simultaneously. To this end, it utilizes a crowd-sourcing approach, not only considering disease-related genes but also borrowing knowledge from similar diseases to infer relevant proteins potentially mediating shared phenotypes. For each disease, GLADIATOR starts from a small set of connected proteins (seed proteins) that are known to associate with that disease, and systematically expands the seeds into connected modules. The expansion aims at minimizing the squared distance between the gold-standard disease similarity to the module similarity, based on the observation that diseases that share common phenotypes are likely to share common molecular mechanisms [14, 17, 18]. A schematic overview of GLADIATOR is given in Fig. 1. The identified modules significantly capture known gene-disease associations and are highly enriched with known biological pathways, allowing GLADIATOR to outperform previous methods.

## Methods

### The GLADIATOR algorithm

The main objective of our method is to identify a collection of disease modules, i.e., a collection of sets of proteins that are connected in the network, whose membership similarity (computed via the Jaccard index) is strongly correlated with a gold-standard phenotypic similarity. We define the membership similarity between two modules (*ModuleSim*) as the size of the intersection between their associated proteins over the size of the union of those sets such that:

$$ ModuleSi{m}_{i, j}= Jaccard\left( Modul{e}_i, Modul{e}_j\right)=\frac{\left|\left| Modul{e}_i{\displaystyle \cap } Modul{e}_j\right|\right|}{\left|\left| Modul{e}_i{\displaystyle \cup } Modul{e}_j\right|\right|} $$

Gold-standard phenotypic similarity was retrieved from the human symptom-disease network [18] and denoted by cosine similarity of the disease-associated symptoms vector as follows:

$$ PhenSi{m}_{i, j}= C o s\left( Phe{n}_i, Phe{n}_j\right)=\frac{{\displaystyle {\sum}_x} Phe{n}_{i, x}, Phe{n}_{j, x}}{\sqrt{{\displaystyle {\sum}_x} Phe{n}_{i, x}^2}\sqrt{{\displaystyle {\sum}_i} Phe{n}_{j, x}^2}} $$

where *Phen*_*i*_ is the vector of symptoms associated with disease *i*. Given the external phenotypic-based similarity (*PhenSim*), our objective is to minimize the sum of squared differences between the two similarities:

1 $$ min\Big({\displaystyle {\sum}_{i< j}{\left( PhenSi{m}_{i, j}- ModuleSi{m}_{i, j}\right)}^2} $$

where *i* and *j* represent disease indices, ranging over the 24,753 disease pairs obtained for the 223 analyzed diseases. We applied a simulated annealing algorithm to traverse the search space of disease-related proteins starting from a connected Seed Protein Set (SeedPS) and expanding it to the final disease module according to the objective function (1). To obtain connected disease modules, we first calculated the largest connected component (LCC) for each disease from its set of Known Disease Protein Set (KnownDisPS) and used it as the initial starting point, or seed, for the annealing process. Ties in the LCC size were broken arbitrarily by selecting the LCC with the smallest index value returned by the connected_components function using the Python NetworkX package. Re-executing GLADIATOR with the set of alternative LCCs of the same sizes returned similar results in terms of the final objective function value and the enrichment of the resulting modules vs. external data sources. KnownDisPS was obtained from [14] (see “Data sources” for full details). Next, in each annealing step we chose a random disease and a random protein to either add or remove. Protein addition was done by choosing a random protein from the set of neighbors available for the current module, while protein removal was done by choosing a random non-seed protein from the current disease module, followed by the removal of additional proteins which were consequently disconnected from the SeedPS. The module similarity matrix was then updated and compared to the gold-standard phenotypic similarity (Eq. (1)), leading to the acceptance or rejection of the module perturbation. The annealing pseudo-code is given in Algorithm 1.
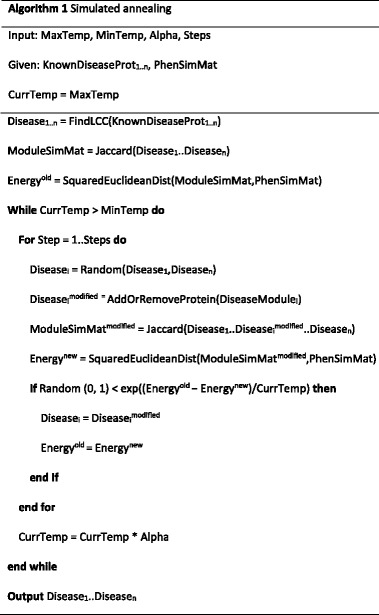


**Fig. 2:**
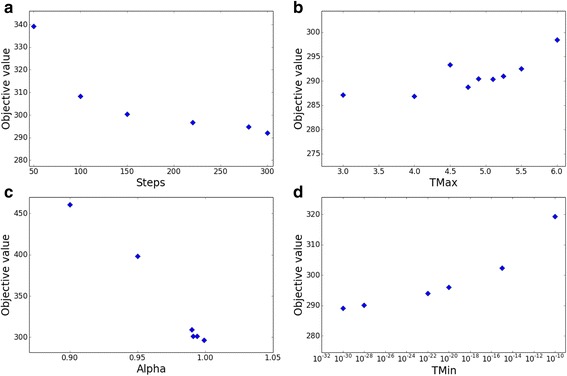
Parameter settings. Final squared Euclidean distance obtained by the algorithm as a function of number of steps to perform at each temperature (**a**), maximal temperature (**b**), temperature decrease rate alpha (**c**), and minimal temperature (**d**)

The annealing procedure takes four parameters: (1) the initial annealing temperature (MaxTemp), (2) the final annealing temperature (MinTemp), (3) the temperature decrease rate (Alpha), and (4) the number of steps to perform in each temperature (Steps). We tested each of these parameters separately while keeping the other three parameters fixed (Fig. 2) and found that for each parameter a tradeoff exists between the objective and running time. For example, when increasing the number of steps, the final difference score decreases, while the running time increases. Moreover, the final score was highly dependent on the cooling schedule. As shown in Fig. 2c, as alpha increases toward 1 (slower cooling), the final energy decreases and reaches saturation around 0.95. However, there was no observable effect of the starting energy on the final results. More importantly, we noticed that the algorithm reaches a saturation point at squared Euclidean distance ≈ 290, after which different parameter configurations increase the running time, while the improvement obtained in the results is negligible (Fig. 2). Based on this analysis, we chose the following parameters: MaxTemp = 5, MinTemp = 1e^-25^, Alpha = 0.995, Steps = 200, balancing between running time and minimal distance obtained. Additional files 1 and 2 demonstrate the robustness of the algorithm to fine tuning of the parameters and random seed, respectively. We tested the GLADIATOR algorithm with 40 different seeds and 25 parameter configurations, obtaining different modules for each run. We found that all runs resulted in similar objective values with an average = 294 ± 3.5 (307 ± 37) for different seed (parameter) configurations. Moreover, all parameter and random seed configurations yielded highly enriched modules compared to known disease-associated genes obtained from DisGeNET [26] (see ”Performance evaluation’), with enrichment vs. ‘Curated’ ranging between 8.1e^-58^ to 3.5e^-85^ for different seed configurations, and between 1.7e^-26^ to 3.2e^-89^ for different parameter configurations; see Additional files 1 and 2. The source code for GLADIATOR is given in Additional file 3 and also can be downloaded from http://www.cs.tau.ac.il/~roded/GLADIATOR.zip.

### Performance evaluation

To evaluate the agreement of the predicted modules with current knowledge, we performed three types of tests. First, cross-validation was performed by repeating the following process on each disease separately. The disease’s SeedPS was pruned by randomly selecting a protein and removing it and all subsequently disconnected proteins until at least 10% of the original SeedPS proteins were removed. Subsequently, GLADIATOR was executed, and the recovery rate for this cross-validation set was evaluated. Diseases with a SeedPS of size 1 were dismissed from the analysis, resulting in 209 diseases tested with an average of 30% of the association removed in each disease and a total of 17% of disease-SeedPS associations tested across all diseases. We repeated the cross-validation test with the pruned SeedPS obtained by this procedure to evaluate the recovery rate of the competing methods.

Next, we evaluated the recovery rate of KnownDisPS which were not a part of the LCC forming the SeedPS in the predicted modules.

Last, we compared the association between Predicted Protein Sets (PredictedPS) and their corresponding diseases to an external data source of disease-gene association obtained from DisGeNET. PredictedPS was obtained from the resulting Modules Protein Set (ModulePS) excluding the SeedPS. Three types of disease-gene associations exist in DisGeNET: (1) associations extracted from the literature (specifically associations extracted from BeFree, the Genetic Association Database (GAD), and the Literature-derived Human Gene-Disease Network (LHGDN)), termed ‘Literature’; (2) curated associations extracted from UniProt and the Comparative Toxicogenomics Database (CTD), termed ‘Curated’; and (3) associations obtained by text mining MEDLINE abstracts using the BeFree system, termed ‘BeFree’, representing a subset of the literature associations. For each association type we constructed a gold-standard association matrix of disease proteins. Each association matrix included diseases found in both the DisGeNET data source and in our ground set of diseases and proteins available in DisGeNET and in the PPI network (see Data sources). Overall, we obtained 5486 associations for 204 diseases in the ‘Curated’ matrix, 57,496 associations for 215 diseases in the ‘Literature’ matrix, and 45,576 associations for 194 diseases in the ‘BeFree’ matrix, with an average of 26.9, 267.4, and 234.9 associations per disease, respectively.

Next, to evaluate the biological attributes of the predicted modules, we examined their functional coherence with respect to the Gene Ontology (GO) and their enrichment with known biological pathways. To compare the predicted modules to known biological pathways, we downloaded pathway annotations from the Molecular Signatures Database (MSigDB) [27], which integrates pathway annotations from multiple data sources. Out of 4726 pathways, we focused on 674 pathways obtained from Reactome [28], 186 pathways obtained from the Kyoto Encyclopedia of Genes and Genomes (KEGG) [29], and 217 pathways obtained from BioCarta. For each pathway we computed the hypergeometric enrichment score for the SeedPS, KnownDisPS, and ModulePS. Next, a false discovery rate (FDR) correction was performed for each disease separately vs. all pathways. To evaluate the coherence of the predicted modules with the enriched pathways in the SeedPS or KnownDisPS, we counted the number of proteins in the PredictedPS which participated in an enriched pathway in the SeedPS or KnownDisPS. We then performed hypergeometric enrichment for the number of true hits in the space of PredictedPS, and the number of proteins participating in all SeedPS or KnownDisPS enriched pathways.

GO [23] was used to examine the biological coherence of our modules. The GO annotations were downloaded in March 2016. To avoid circularity, we eliminated annotations inferred from physical interactions (evidence coded inferred from physical interaction (IPI)). We used the R package csbi [30] to calculate semantic similarity scores between all protein pairs according to the Resnik similarity metric [31]. The similarity score was calculated for biological process, molecular function, and cellular component separately, resulting in three protein-similarity matrices. To evaluate significance, we constructed for each disease 100 random modules, starting from the same disease SeedPS, and while keeping the module connected, we randomly added or removed proteins until the module size reached the predicted size by GLADIATOR for that disease. Protein addition was done from the set of neighbors available for the expanding module, while protein removal was done by removing a random protein from the module as well as all proteins which were disconnected from the SeedPS as a result. Empirical *p* values were obtained by comparing the average Resnik similarity of the predicted ModulePS to the distribution score of the average Resnik similarity across the 100 random modules for the corresponding disease.

### Comparison to previous methods

We compare our method to two current state-of-the-art methods: the DIAMOnD algorithm and HotNet2. DIAMOnD takes four inputs: network, seed proteins, desired number of DIAMOnD proteins, and seed weights. We evaluated DIAMOnD by using the same network and two forms of seed proteins: one considering KnownDisPS and the other considering only SeedPS. The seed weight was set to the default value as suggested by the authors. The number of desired proteins in each module was set, for comparison purposes, to the number of PredictedPS obtained by GLADIATOR for that disease.

HotNet2 requires an initial heat score for all proteins in the graph. Following Mazza et al. [13], we tested two configurations of initial input heat for SeedPS, InitHeat = 100 and 1000, and assigned a heat of 1 to all other proteins. A subnetwork produced by HotNet2 was considered significant if the empirical *p* value reported for it was smaller than 0.05. As only a small fraction of diseases achieved significant modules (32 diseases for InitHeat = 1000 and 17 for InitHeat = 100), we considered two variances of the HotNet2 solution, one considering only the significant modules predicted for a subset of diseases and the second when considering the largest obtained module for all diseases regardless of its significance score. We thus obtained four variants of HotNet2, termed HotNet2, Heat 1000/100, and Significant/All.

The average module size obtained by HotNet2 for input InitHeat = 1000 was 72.5 and 144.4 proteins per module, considering only significant modules and all modules, respectively. When altering the initial input heat to 100, the numbers increased to 118.5 and 197.4, respectively. The enrichment scores reported in the Results section correspond to InitHeat = 1000. The comparison to all four variants of HotNet2 is given in Additional file 4: Figure S1.

### Data sources

Disease-gene associations were retrieved from Menche et al. [14]; this work focuses on associations obtained from the Mendelian Inheritance in Man (OMIM) [1] and the Genome-Wide Association Study (GWAS) Catalog [2] databases, resulting in a corpus of 299 diseases. Disease-related phenotypes and phenotypic similarity were obtained from Zhou et al. [18]. Overall, disease-phenotypic similarity was obtained for 1596 diseases from which 223 existed in our dataset, enabling the application of our method to this disease set. Finally, we used a comprehensive set of protein-protein interactions obtained from [14], which was compiled from 15 data sources including regulatory interactions [32], binary protein interactions (e.g., [33]), metabolic interactions [34], complex interactions [35], kinase interactions [36], signal interactions [37], and curated interactions (e.g., [38]); see [14] for full details.

## Results

### Overview of GLADIATOR

We devised a novel method for inferring disease modules for multiple diseases simultaneously. It is based on inferring modules so that the resulting module similarity is as close as possible to a given phenotypic disease-disease similarity. To motivate this approach, we tested the correlation between disease phenotypic similarity and disease genetic similarity as reflected in our dataset. Phenotypic similarity was retrieved from the human symptoms-disease network [16] and represents the cosine similarity between the vectors of disease-associated phenotypes (see “Methods”). The “genetic” module similarity was defined using a Jaccard score, which is the number of genes shared by the disease modules over the size of the union of these sets. Disease-related proteins (referred to throughout as Known Disease Protein Set, or KnownDisPS) were obtained from the OMIM and GWAS catalogs (Methods). The genetic similarity was strongly correlated with the phenotypic similarity with a Pearson correlation of 0.28 (*p* value = 0, see Additional file 4: Figure S2).

**Fig. 3:**
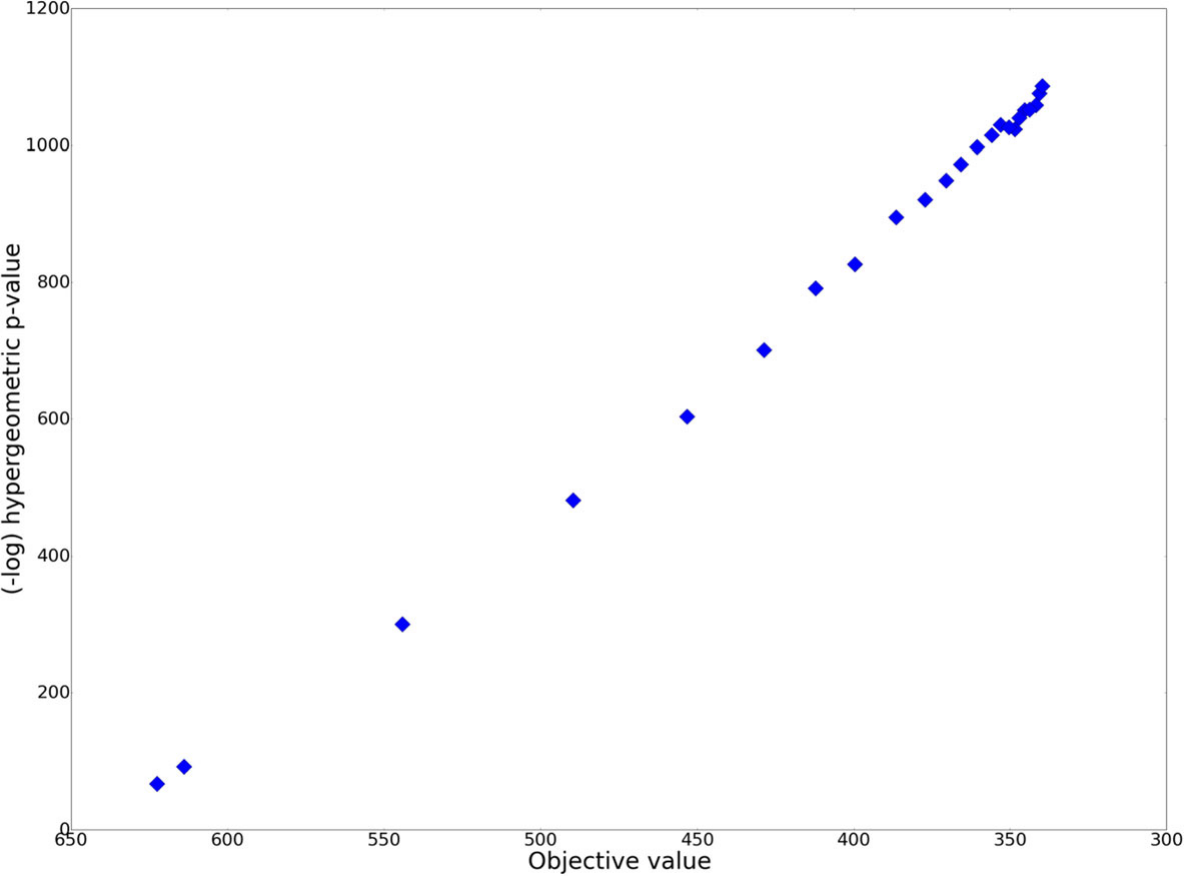
The objective function guides detection of modules enriched with known disease-related proteins. As the annealing process minimizes the Euclidean distance between module similarity and phenotypic similarity, the disease modules become more enriched with disease-related proteins, measured by the –log hypergeometric *p* value between associations predicted by GLADIATOR’s modules and gold-standard associations retrieved from DisGeNET

Our method, named GLADIATOR (GLobal Approach for DIsease AssociaTed mOdule Reconstruction), initializes Seed Protein Sets (referred to as SeedPS) and expands them to complete modules by minimizing the squared distance between the given disease phenotypic similarity and the similarity of the corresponding modules in terms of their member proteins (see Fig. 1). SeedPS was set for each disease as a set of proteins forming the largest connected component (LCC) out of all the connected components spanned by the KnownDisPS in the human PPI network. To traverse the search space of potential disease-related proteins, we applied a simulated annealing algorithm targeted at optimizing the correlation between the phenotypic- and module-based disease similarities. In each step of the annealing process, we randomly selected a disease-protein pair to either add or remove, while ensuring the connectivity of the resulting output module (Methods). We confirmed that this process converges to coherent modules by plotting the hypergeometric enrichment score of the intermediate modules as a function of the objective function score as the annealing progresses. As shown in Fig. 3, the enrichments of the predicted proteins increase as the distance between module similarity and phenotypic similarity decreases, reinforcing our approach.

The GLADIATOR algorithm inferred 223 disease modules with an average Module Protein Set (referred to as ModulePS) size of 47.3 proteins, a Pearson correlation to phenotypic similarity of 0.68, and a squared Euclidean distance of 291.5. This result shows great improvement over the initial starting point of the modules, with an average module size of 18.1 and a Pearson correlation of 0.2 to phenotypic similarity for the SeedPS, and an average size of 64.3 proteins with a Pearson correlation of 0.28 for the KnownDisPS. The resulting disease modules are provided in Additional file 5. Overall, GLADIATOR was able to expand SeedPS, resulting in a total of 6497 new disease-protein associations for 2373 proteins and 216 diseases. The predicted proteins vary in their topological properties such as degree and centrality. The average degree of the predicted proteins was higher than expected by chance, as was also observed previously for known disease-associated proteins [12]; see Additional file 4: Figure S3. The predicted proteins were highly enriched in known disease-related proteins and in relevant cellular pathways, as demonstrated below.

### GLADIATOR predicts known disease-related genes

In order to evaluate the correlation between disease modules predicted by GLADIATOR and the current knowledge base of disease-associated genes, we employed two types of cross-validation tests and additionally compared the predicted associations to an external data source of disease-gene association. First, we examined the recovery rate of known disease proteins from the OMIM and GWAS catalogs which were not part of the LCCs that served as seed sets for the module reconstruction (i.e., KnownDisPS excluding SeedPS). Overall, the KnownDisPS for all diseases involves 14,338 known disease-protein associations, 4041 of which were in SeedPS. Out of the 6497 associations predicted by GLADIATOR, 301 true associations were recovered, resulting in a highly significant hypergeometric *p* value < 6e^-186^. Furthermore, when analyzed separately, 101 diseases (45%) were enriched with known disease proteins with a hypergeometric FDR-corrected *p* value < 0.05 (Methods). Additionally, a cross-validation test was applied to each disease separately by removing at least 10% of its original SeedPS, while ensuring the seed’s connectivity (Methods). Overall, 127 disease-gene associations out of 640 associations removed (19.8%) were recovered across all disease modules.

Next, we compared the novel Predicted Protein Set, PredictedPS (i.e., ModulePS excluding SeedPS) to an external corpus of disease-gene associations extracted from the DisGeNET database [26]. We constructed a gold-standard disease-gene association matrix for the three types of associations obtained from DisGeNET, termed here as ‘Literature’, ‘Curated’, and ‘BeFree’ (Methods). We computed hypergeometric enrichments for associations predicted by GLADIATOR across all diseases vs. gold-standard drug-gene associations (after removing SeedPS). The predicted associations showed significant enrichments with all association types (*p* values < 5e^-491^, 2e^-543^, and 5e^-83^ for ‘Literature’, ‘BeFree’, and ‘Curated’ respectively). We further computed the per-disease enrichment for each PredictedPS vs. the corresponding gold-standard disease associations. We found that 34%, 7.5%, and 34% of the predicted modules were significantly enriched when compared against the ‘Literature’, ‘Curated’, and ‘BeFree’ associations, respectively (FDR-corrected hypergeometric *p* value < 0.05). A total of 103 out of 214 diseases (48%) with available gold-standard associations were significantly enriched in at least one of the association types. Overall, 151 modules (68%) were validated with gold-standard associations extracted from either DisGeNET or the OMIM and GWAS catalogs (KnownDisPS).

### GLADIATOR predicts coherent modules

To evaluate the biological properties of the modules predicted by GLADIATOR, we examined both their functional coherence with respect to the GO and their enrichment with known biological pathways. First, we calculated the average semantic similarity for each ModulePS in GO biological process (BP), molecular function (MF), and cellular component (CC) categories. We compared the resulting scores to the coherence scores of 100 random modules obtained by randomly expanding each SeedPS to a connected module of the same size as the original module (Methods). We found that 34.4%, 56.1%, and 64.7% of the predicted modules were highly coherent in CC, MF, and BP terms, respectively (empirical *p* value < 0.05), with 70% of the modules exhibiting significant coherence in at least one of the three GO branches. For the second test, the ModulePS were tested against each of the 1077 gold-standard pathways retrieved from MSigDB using a hypergeometric test. We found that 216 disease modules (97%) predicted by GLADIATOR were enriched in at least one known biological pathway (FDR *p* value <0.05, corrected for modules and pathways) with an average of 75 enriched pathways per ModulePS (FDR-corrected *p* value < 0.05). To evaluate the significance of these results, we constructed 100 randomized models. Each such model was composed from a collection of random expansions of each disease SeedPS to a connected module of the same size as the corresponding GLADIATOR module. On average, 200 modules per random model were enriched in at least one pathway (FDR *p* value < 0.05) with an average of 22 enriched pathways per module, resulting in an empirical *p* value < 0.01 compared to GLADIATOR in both tests. We further computed the enrichment of SeedPS and KnownDisPS in MSigDB pathways, collecting the significantly enriched pathways (FDR <0.05) in these protein sets to a reference collection of pathways. The average number of enriched pathways in SeedPS and KnownDisPS was 23.8 and 25.3 pathways, respectively, representing a fivefold-to-fourfold decreased enrichment vs. the ModulePS. Furthermore, 91% of the SeedPS reference pathways remained enriched in the corresponding ModulePS.

Last, to evaluate the relevance of the novel predicted proteins to disease etiology, we compared the pathway annotation of PredictedPS to the reference pathways enriched in the corresponding SeedPS (Methods). We found that 77% of diseases’ PredictedPS were significantly enriched in proteins participating in SeedPS reference pathways, validating their relevance. Moreover, 83% of the PredictedPS were validated in a similar manner when compared to the reference pathways enriched in the KnownDisPS (Methods).

### GLADIATOR outperforms existing methods

We compared our method to two recently published state-of-the-art methods for predicting disease-associated modules: DIAMOnD [15] and HotNet2 [16]. We applied the DIAMOnD algorithm to the set of diseases, using either the SeedPS used by the GLADIATOR algorithm or the entire set of KnownDisPS from which the SeedPS was extracted. DIAMOnD iteratively adds proteins to a set of seed proteins until the disease module size meets a predefined target size. For comparison, we fixed DIAMOnD’s target size parameter to the module size obtained by the GLADIATOR algorithm for the same disease. A module returned by the HotNet2 algorithm was considered significant if the empirical *p* value reported for its size was less than 0.05. Out of the 223 modules predicted by HotNet2 for all diseases, only 32 (14%) were reported as significant according to their empirical *p* value. We thus considered two HotNet2 solutions: one containing all 223 modules, regardless of their empirical *p* value, and another containing only the 32 modules reported as significant by HotNet2 (Methods).

**Fig. 4:**
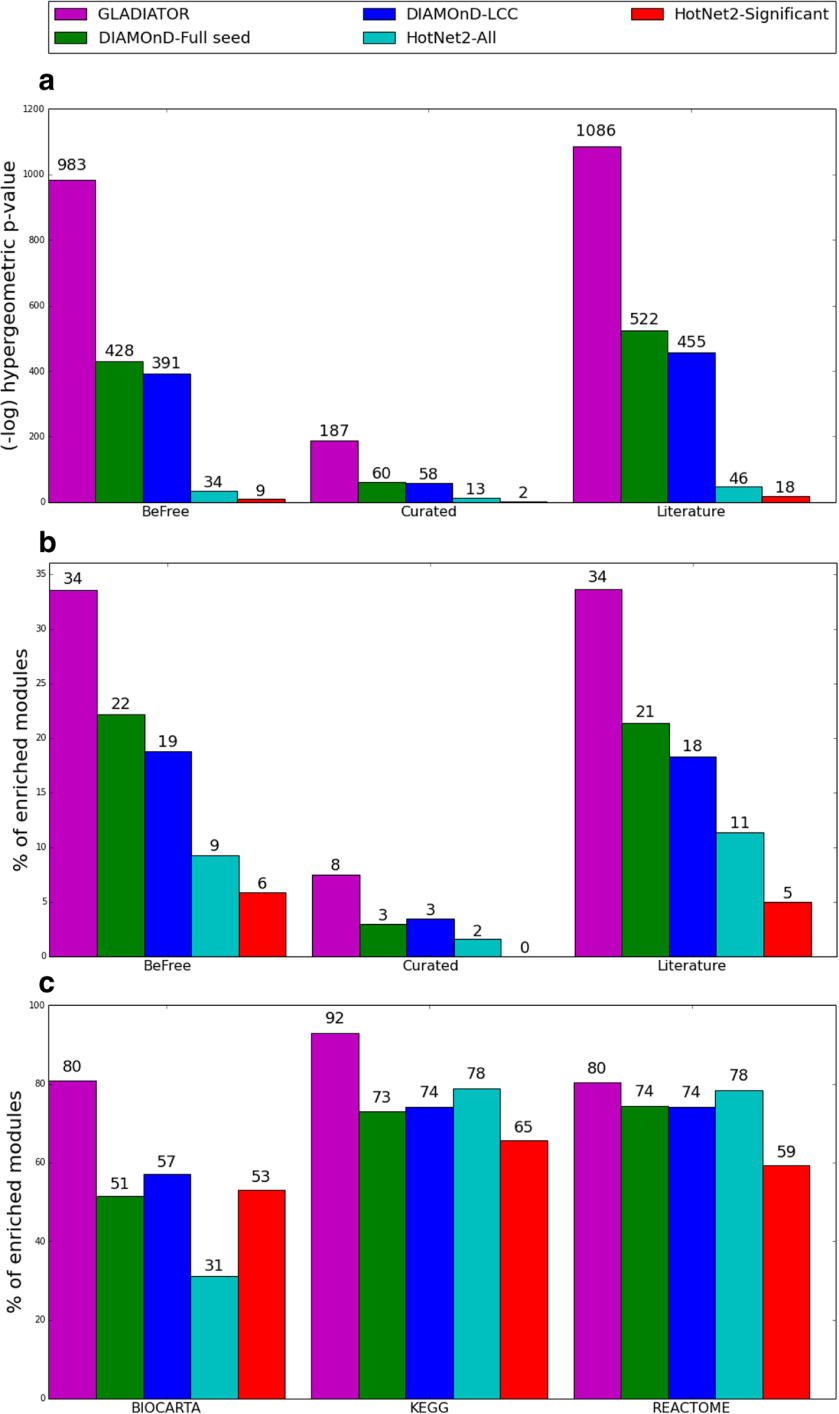
GLADIATOR outperforms previous methods. Enrichment scores (–log(hypergeometric *p* value)) for disease-gene associations extracted from DisGeNET vs. all predicted disease-gene associations (**a**); percentage of enriched modules vs. corresponding disease gold-standard associations extracted from DisGeNET (**b**); percentage of enriched modules vs. known pathways extracted from MSigDB (**c**). GLADIATOR predictions were compared to the modules obtained from the DIAMOnD algorithm, using both the full list of disease-gene associations (*DIAMOnD-Full seed*) and the largest connected component obtained from this list, which served as the seed for our algorithm (*DIAMOnD-LCC*), and to the largest modules obtained from the HotNet2 algorithm (*HotNet2-All*) and a subset of these modules reported as significant by the HotNet2 algorithm (*HotNet2-Significant*)

We repeated all evaluation tests on the competing methods. In cross-validation, DIAMOnD and HotNet2 were able to recover 13.3% and 9.9% of the removed associations, when starting from the same pruned SeedPS as GLADIATOR, compared to 19.8% associations retrieved by GLADIATOR. Moreover, accounting for KnownDisPS associations, DIAMOnD was able to recover only 24 associations out of its 6502 predicted associations (hypergeometric *p* value of 0.89), while HotNet2 was able to recover 195 associations out of its 40,829 predicted associations (hypergeometric *p* value of 0.51) compared to 301 out of the 6497 associations predicted by GLADIATOR (hypergeometric *p* value < 6e^-186^). Figure 4 depicts the performance of GLADIATOR, HotNet2, and DIAMOnD with respect to gold-standard disease-gene associations and biological pathway enrichments. Evidently, GLADIATOR outperforms both methods, obtaining the highest enrichment in all three association types extracted from DisGeNET and retaining the highest percentage of predicted modules enriched with biological pathways or known associations.

### GLADIATOR predicts novel shared proteins among phenotypically similar diseases

One desired property of GLADIATOR is its ability to predict shared submodules among similar diseases. Overall, 6617 disease pairs (out of 24,753 pairs) from our dataset have a positive phenotypic similarity score. Out of these, 3147 disease pairs showed genetic similarity (i.e., shared genes) in their KnownDisPS, and only 884 pairs showed genetic similarity in their SeedPS. GLADIATOR was able to recover genetic similarity for 5652 disease pairs out of the phenotypically similar ones. Additional file 4: Figure S2 displays the correlation between genetic similarity and phenotypic similarity before and after running GLADIATOR.

Disease module similarity assigned to a disease pair which did not exhibit genetic similarity in its SeedPS results from introducing previously unknown shared proteins to two similar diseases or by adding a novel protein to a disease, where it is already known to associate with a similar disease. Doing so, GLADIATOR expanded the SeedPS with 1031 shared proteins, 105 of which were not previously associated with any disease. The histogram of associated diseases per protein is shown in Additional file 4: Figure S3c. Out of the 301 known disease proteins recovered by GLADIATOR, 284 were recovered by assigning a seed protein from one disease to a phenotypically similar disease. For example, oligodendrocyte transcription factor (OLIG2) has a known role in both leukemia and in lymphatic diseases, which have a phenotypic similarity of 0.6. OLIG2 was part of the SeedPS of leukemia; however, it was not part of the LCC that served as SeedPS in lymphatic diseases. OLIG2 was predicted by GLADIATOR to participate in the lymphatic diseases module along with 29 more proteins, resulting in a ModulePS of size 103. In the final predicted ModulePS, lymphatic diseases and leukemia share 48 proteins, compared to 67 in their KnownDisPS and 19 in their SeedPS.

As another example, we analyzed a common protein predicted to have a role in both autoimmune diseases and blood platelet disorders, which have a phenotypic similarity of 0.7. These two diseases, which share no proteins in their SeedPS and only two proteins in their KnownDisPS, were assigned with 30 shared proteins in their ModulePS, 14 of which were not previously known to associate with any of the diseases. Fibrinogen gamma chain (FGG) is one example of such a protein. FGG is a blood-borne glycoprotein which upon vascular injury is cleaved to form fibrin. Both fibrinogen and its cleavage product fibrin have multiple functions in blood clotting, including platelet aggregation [39, 40]. Specifically, the binding of fibrinogen through its gamma chain (FGG) allows platelet aggregation and wound healing. Variations in this binding site were shown to contribute to disorders such as thrombosis and cardiovascular disease [41]. Furthermore, inhibition of gamma chain function has been shown to interfere with multiple fibrinogen activities, including platelet adhesion and platelet-mediated clot retraction [41]. Recently it was shown that in several autoimmune neurodegenerative diseases, such as multiple sclerosis, disruption in the blood-brain barrier allows fibrinogen to contact the white matter, which forms autoimmunogenic fibrin plaques [42]. Additionally, it was shown that fibrinogen promotes autoimmunity via chemokine release [43] and that an abnormal variant in fibrinogen occurs in patients with certain types of autoimmune diseases [44].

### Case analysis: the hyperinsulinism module

**Fig. 5:**
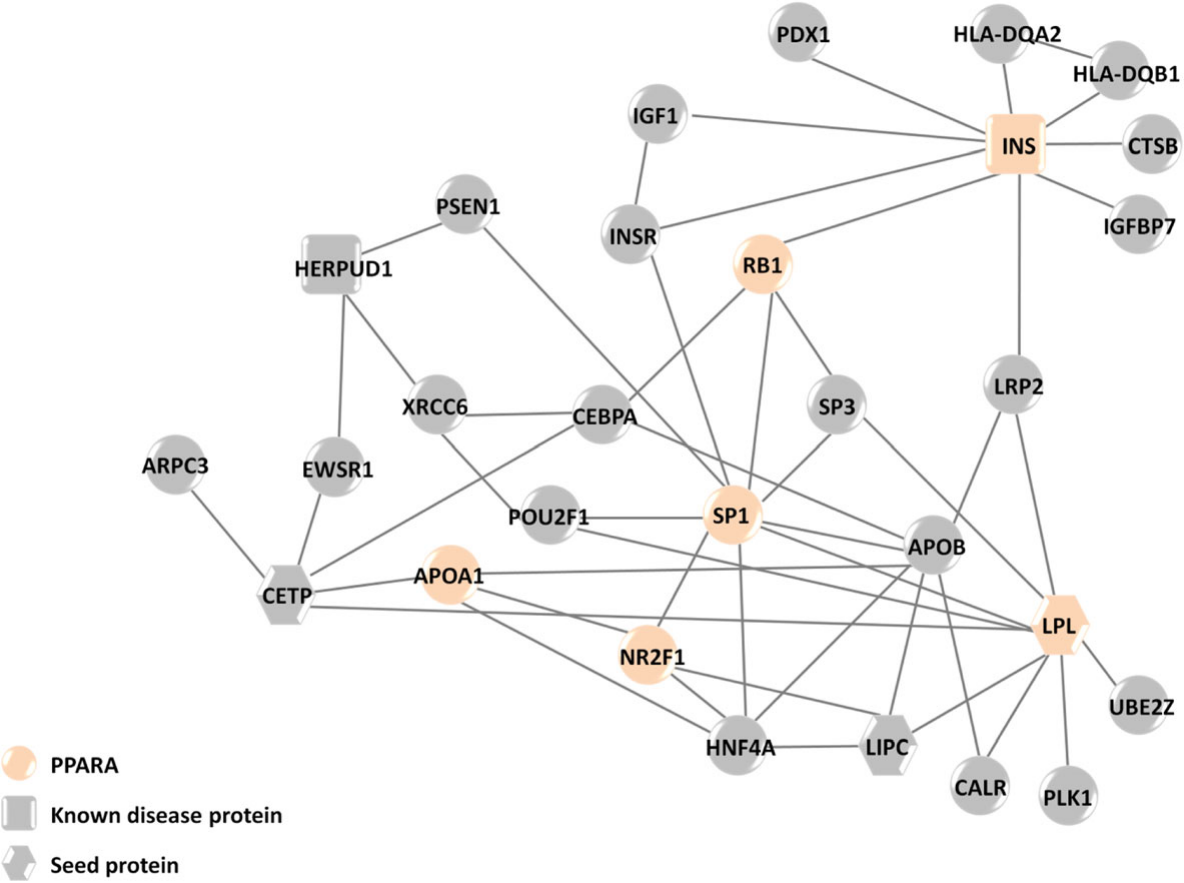
The disease module predicted by GLADIATOR for hyperinsulinism. Proteins participating in the enriched peroxisome proliferator-activated receptor alpha (*PPARA*) pathway are colored accordingly. Proteins participating in SeedPS or KnownDisPS are marked as *hexagons* and *quadrangles*, respectively

After establishing the utility of our method across different diseases, we expanded the analysis on a specific disease module, focusing on hyperinsulinism, a medical state in which the levels of insulin in the blood are above the norm. Only one protein in OMIM is known to be related to hyperinsulinism, while 44 proteins are reported in the GWAS Catalog. Out of these 45 proteins, 26 were found in the PPI network, and only 3 were initially connected and served as SeedPS. GLADIATOR expanded this seed to a module of 29 proteins, from which 2 were recovered from its set of KnownDisPS (Fig. 5). Comparing the ModulePS of hyperinsulinism to known pathways retrieved from MSigDB resulted in 10 enriched pathways (FDR-corrected *p* value < 0.05), 7 of which are also enriched in the PredictedPS, while only 4 are enriched in its SeedPS. In the following section, we focus on one of these pathways and show how it affects disease pathogenicity, by analyzing proteins from this pathway predicted by GLADIATOR to associate with hyperinsulinism, and we further analyze novel suggested genes.

The peroxisome proliferator-activated receptor alpha (PPARA) pathway (BioCarta) was enriched in the predicted ModulePS, with 6 out of its 60 proteins participating in the predicted hyperinsulinism module (see Fig. 5). PPARA is a nuclear transcription factor and a major regulator of lipid metabolism; it regulates the expression of genes involved in lipoprotein metabolism, raising the levels of apolipoprotein A1 (APOA1), a major apolipoprotein of high-density lipoprotein (HDL) [45]. APOA1, predicted by GLADIATOR to participate in the hyperinsulinism module, is well studied in the context of insulin and glucose regulation. It has been long known that APOA1 is a significant contributor to the plasma insulin concentration in men [46], and an inverse correlation between insulin released during an oral glucose tolerance test and APOA1 concentration was observed [47]. Moreover, polymorphism in the APOA1-C3-A4 gene cluster was associated with hyperlipidemia [48] and fasting insulin levels [49]. GLADIATOR also predicted APOB, another apolipoprotein, to participate in the disease module. It was shown that the level of APOB in plasma is a good predictor for both glucose level and incident type 2 diabetes. Moreover, the APOA1/HDL cholesterol ratio was the strongest predictor of incident type 2 diabetes [50], and APOB level is significantly correlated with plasma insulin level in women [46]. Another key protein in the PPARA pathway is the transcription factor specificity protein 1 (SP1). SP1, predicted by GLADIATOR, is involved in several mechanisms altering gene activity in response to insulin [51]. Moreover, it was shown that insulin regulates the subcellular localization, stability, and activation level of SP1 [52]. We noticed that another member of the SP family, SP3, is also predicted by GLADIATOR to participate in the hyperinsulinism module, and found that a recent study suggests a new mechanism involving both SP1 and SP3 in mediating insulin activation of glucokinase transcription [53]. Last, we found that the retinoblastoma protein RB1, which also participates in the PPARA pathway, is associated with insulin resistance, obesity, and metabolic disturbances in mice [54]. We thus suggest that dysregulation of the PPARA pathway may promote hyperinsulinism.

## Discussion

GLADIATOR predicts disease modules for hundreds of diseases simultaneously based on a protein interaction network and disease phenotypic similarity. The predicted modules were compared with external data sources, showing a strong correlation with current knowledge of disease-related genes. Moreover, we tested the coherence of our modules by comparing them both to existing biological pathways and to GO-related terms, obtaining high enrichment and similarity scores. We provided a detailed analysis of the shared molecular mechanism of similar diseases and further investigated the hyperinsulinism module predicted by GLADIATOR, demonstrating its pathophysiological relevance and suggesting possible mechanisms underlying this disease.

Previous module-finding methods were gene-centric or disease-centric and lacked information on the cross-talk between different disease components. In contrast, the module-centric approach presented here provides a wider, integrated view. Given that many human diseases share mechanisms and phenotypes, we hypothesize that knowledge obtained for one can be borrowed to infer mechanisms for other similar diseases. GLADIATOR provides not only predicted modules but also the inter-relation between disease modules, in the form of shared submodules, providing insights into the etiology and phenotypic mechanisms of diseases. We demonstrate that indeed as the correlation between module similarity and the phenotypic similarity increases, the modules become more predictive to known disease-related proteins, supporting the assumption that phenotypically similar diseases share similar mechanisms.

In this endeavor we focused on a set of predicted modules viewed as complete disease pathways. However, running GLADIATOR multiple times under different parameter configurations may yield different predicted modules in each run. These modules can then be used to rank proteins according to the percentage of solutions in which they are associated with a disease, thus increasing the robustness of the predictions for both disease-related protein and phenotypic-related submodules. Last, this study focused on finding one connected module for each disease. A future study can expand the understanding and cataloging of diseases mechanisms by utilizing our method as a starting point for multiple pathway predictions per disease.

## Conclusions

We have taken a global approach for predicting hundreds of disease modules simultaneously based on the phenotypic similarity between diseases. Our method utilizes the protein interaction network to find connected regions that form coherent modules which mediate disease pathology. The analysis of the resulting modules demonstrated that borrowing knowledge from one disease can contribute to the molecular understanding of another disease.

## Additional files

Additional file 1: GLADIATOR performance under different parameter configurations. (XLSX 10 kb)

Additional file 2: GLADIATOR performance under different random seeds. (XLSX 11 kb)

Additional file 3: GLADIATOR code. (PY 16 kb)

Additional file 4: Supplementary figures and legends. **Figure S1.** Comparison to HotNet2 variants. Enrichment scores for disease-gene associations extracted from DisGeNet vs. all predicted disease-gene associations (a). Precentage of enriched modules vs. corresponding disease gold standard associations extracted from DisGeNet (b). Precentage of enriched modules vs. known pathways extracted from MSigDB (c). GLADIATOR predictions were compared to four variant of HotNet2 solutions corresponding to two different Heat parameters of 1000 and 100, and two significance thresholds for module of 0.05 and 1. **Figure S2.** Correlation between phenotypic and genetic similarity. Phenotypic similarity vs. Jaccard-based similarity obtained from known diseases associated proteins (KnownDisPS) (a). Seed proteins used by GLADIATOR (SeedPS) (b). Modules predicted by GLADIATOR (ModulePS) (c). The average phenotypic similarity as a function of number of shared proteins between disease pair’s KnownDisPS (d). **Figure S3.** Topological properties of the predicted proteins. Distribution of predicted proteins degree in PPI network (a). Distribution of nodes betweenness centrality for predicted proteins in PPI network (b). Distribution of number of diseases associated with proteins from predicted ModulePS (c). (DOCX 384 kb)

Additional file 5: Disease modules predicted by GLADIATOR algorithm. (XLSX 93 kb)
