# Supplementary material for: GLADIATOR: a global approach for elucidating disease modules
Source: Genome Med. 2017 May 26;9:48. doi: 10.1186/s13073-017-0435-z (PMC5446740; doi:10.1186/s13073-017-0435-z)

**SUPPLEMENTAL FIGURE LEGENDS**

**Figure S1. Comparison to HotNet2 variants.** Enrichment scores for disease-gene associations extracted from DisGeNet vs. all predicted disease-gene associations (a). Precentage of enriched modules vs. corresponding disease gold standard associations extracted from DisGeNet (b). Precentage of enriched modules vs. known pathways extracted from MSigDB (c). GLADIATOR predictions were compared to four variant of HotNet2 solutions corresponding to two different Heat parameters of 1000 and 100, and two significance thresholds for module of 0.05 and 1.

**Figure S2. Correlation between phenotypic and genetic similarity.** Phenotypic similarity vs. Jaccard-based similarity obtained from known diseases associated proteins (KnownDisPS) (a). Seed proteins used by GLADIATOR (SeedPS) (b). Modules predicted by GLADIATOR (ModulePS) (c). The average phenotypic similarity as a function of number of shared proteins between disease pair’s KnownDisPS (d).

**Figure S3**. **Topological properties of the predicted proteins.** Distribution of predicted proteins degree in PPI network (a). Distribution of nodes betweenness centrality for predicted proteins in PPI network (b). Distribution of number of diseases associated with proteins from predicted ModulePS (c).


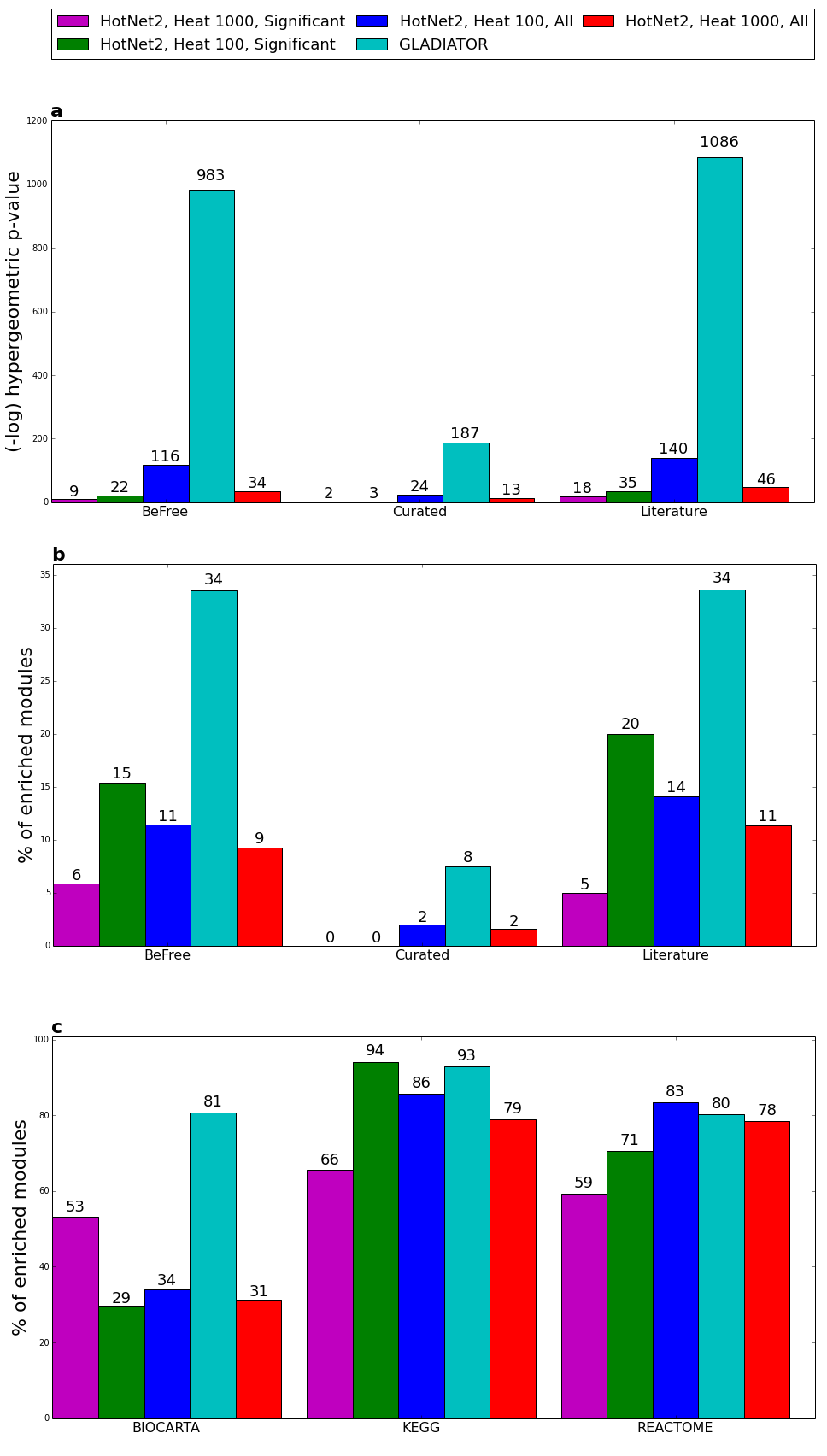


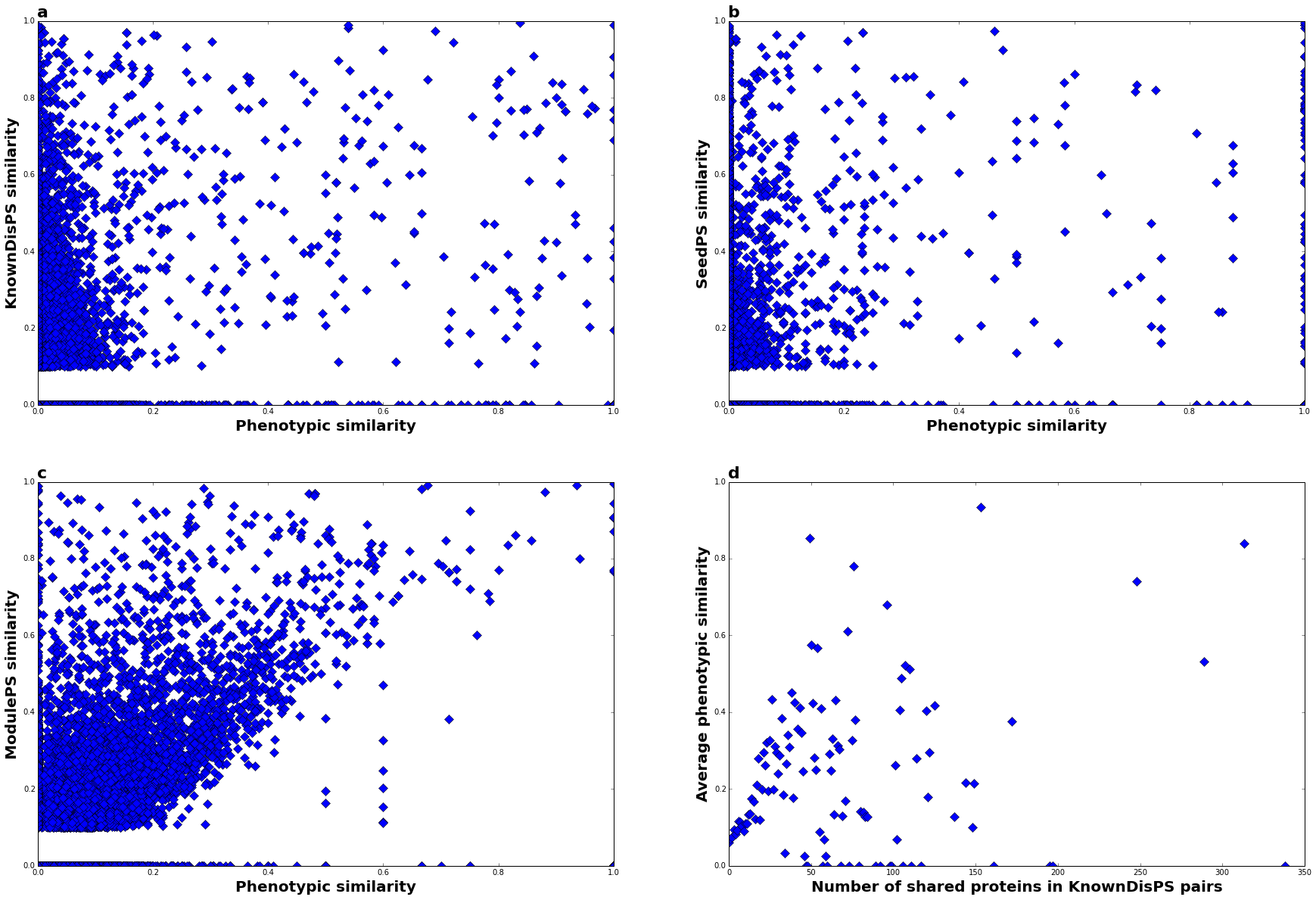


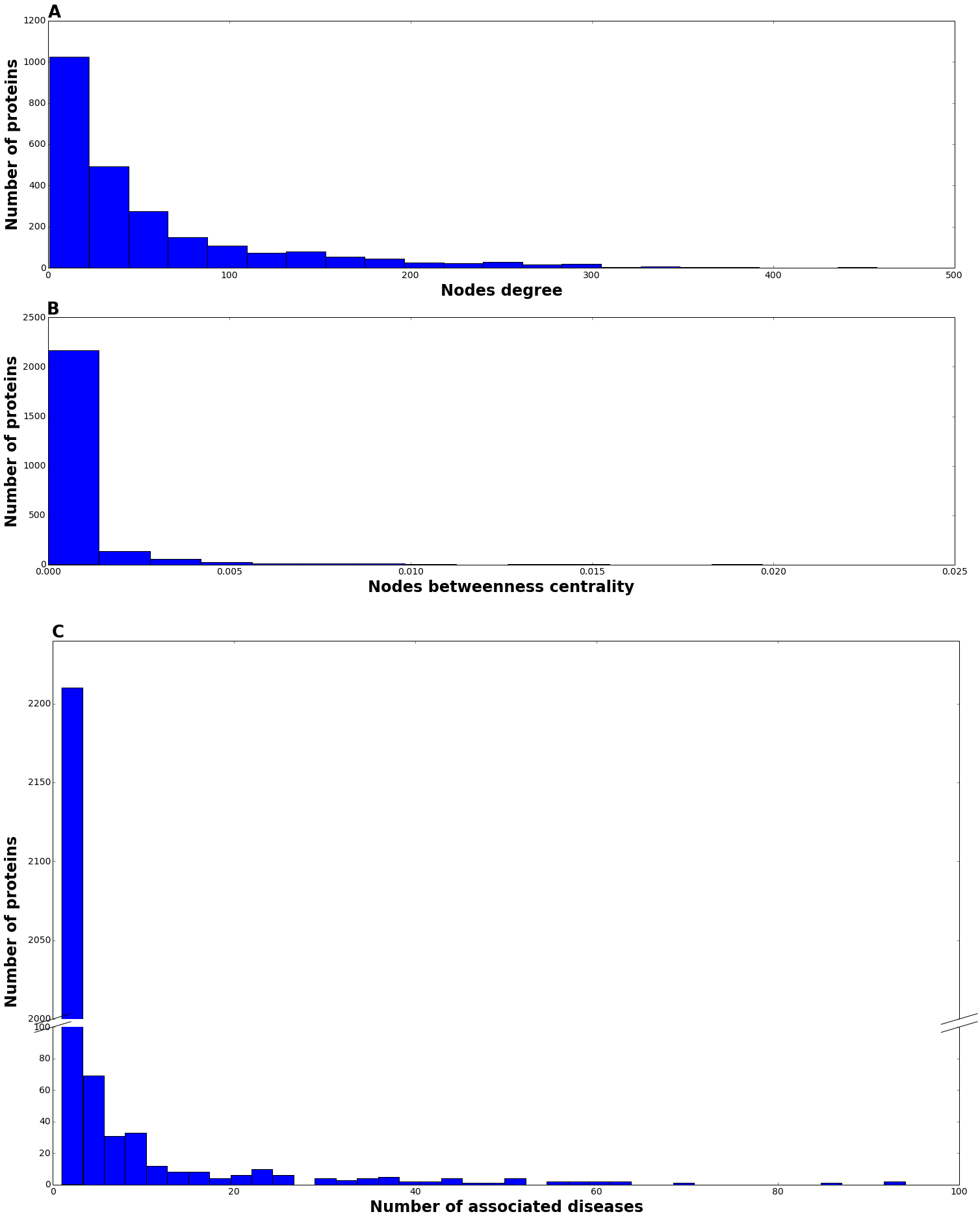

Supplement: Supplementary file 4 — Supplementary figures and legends. Figure S1. Comparison to HotNet2 variants. Enrichment scores for disease-gene associations extracted from DisGeNet vs. all predicted disease-gene associations (a). Precentage of enriched modules vs. corresponding disease gold standard associations extracted from DisGeNet (b). Precentage of enriched modules vs. known pathways extracted from MSigDB (c). GLADIATOR predictions were compared to four variant of HotNet2 solutions corresponding to two different Heat parameters of 1000 and 100, and two significance thresholds for module of 0.05 and 1. Figure S2. Correlation between phenotypic and genetic similarity. Phenotypic similarity vs. Jaccard-based similarity obtained from known diseases associated proteins (KnownDisPS) (a). Seed proteins used by GLADIATOR (SeedPS) (b). Modules predicted by GLADIATOR (ModulePS) (c). The average phenotypic similarity as a function of number of shared proteins between disease pair’s KnownDisPS (d). Figure S3. Topological properties of the predicted proteins. Distribution of predicted proteins degree in PPI network (a). Distribution of nodes betweenness centrality for predicted proteins in PPI network (b). Distribution of number of diseases associated with proteins from predicted ModulePS (c). (DOCX 384 kb) [file 13073_2017_435_MOESM4_ESM.docx]
